# CRISPR-Cas Systems-Based Bacterial Detection: A Scoping Review

**DOI:** 10.3390/diagnostics12061335

**Published:** 2022-05-27

**Authors:** Kasturi Selvam, Mohamad Ahmad Najib, Muhammad Fazli Khalid, Mehmet Ozsoz, Ismail Aziah

**Affiliations:** 1 Institute for Research in Molecular Medicine (INFORMM), Health Campus, Universiti Sains Malaysia, Kubang Kerian 16150, Kelantan, Malaysia; kasturiselvam0612@gmail.com (K.S.); najib@student.usm.my (M.A.N.); fazlikhalid@usm.my (M.F.K.); mehmet.ozsoz@neu.edu.tr (M.O.); 2 Department of Biomedical Engineering, Near East University, Nicosia 99138, Turkey

**Keywords:** bacterial infections, CRISPR, Cas enzymes, detection, scoping review

## Abstract

Recently, CRISPR-Cas system-based assays for bacterial detection have been developed. The aim of this scoping review is to map existing evidence on the utilization of CRISPR-Cas systems in the development of bacterial detection assays. A literature search was conducted using three databases (PubMed, Scopus, and Cochrane Library) and manual searches through the references of identified full texts based on a PROSPERO-registered protocol (CRD42021289140). Studies on bacterial detection using CRISPR-Cas systems that were published before October 2021 were retrieved. The Critical Appraisal Skills Programme (CASP) qualitative checklist was used to assess the risk of bias for all the included studies. Of the 420 studies identified throughout the search, 46 studies that met the inclusion criteria were included in the final analysis. Bacteria from 17 genera were identified utilising CRISPR-Cas systems. Most of the bacteria came from genera such as *Staphylococcus*, *Escherichia*, *Salmonella*, *Listeria*, *Mycobacterium* and *Streptococcus*. Cas12a (64%) is the most often used Cas enzyme in bacterial detection, followed by Cas13a (13%), and Cas9 (11%). To improve the signal of detection, 83% of the research exploited Cas enzymes’ trans-cleavage capabilities to cut tagged reporter probes non-specifically. Most studies used the extraction procedure, whereas only 17% did not. In terms of amplification methods, isothermal reactions were employed in 66% of the studies, followed by PCR (23%). Fluorescence detection (67%) was discovered to be the most commonly used method, while lateral flow biosensors (13%), electrochemical biosensors (11%), and others (9%) were found to be less commonly used. Most of the studies (39) used specific bacterial nucleic acid sequences as a target, while seven used non-nucleic acid targets, including aptamers and antibodies particular to the bacteria under investigation. The turnaround time of the 46 studies was 30 min to 4 h. The limit of detection (LoD) was evaluated in three types of concentration, which include copies per mL, CFU per mL and molarity. Most of the studies used spiked samples (78%) rather than clinical samples (22%) to determine LoD. This review identified the gap in clinical accuracy evaluation of the CRISPR-Cas system in bacterial detection. More research is needed to assess the diagnostic sensitivity and specificity of amplification-free CRISPR-Cas systems in bacterial detection for nucleic acid-based tests.

## 1. Introduction

Bacterial infection occurs when bacteria enter the body, multiply, and cause a reaction in the body. Many patients with suspected bacterial infections are given empiric antimicrobial medicine instead of proper treatment, which leads to an increase in antimicrobial resistance [1]. The ESKAPE bacteria (*Enterococcus faecium*, *Staphylococcus aureus*, *Klebsiella pneumoniae*, *Acinetobacter baumanii*, *Pseudomonas aeruginosa*, and *Enterobacteriaceae*) are the microorganisms that are primarily involved in the resistance process, emphasising their ability to “escape” from common antibacterial treatments [2]. Antibiotic resistance pathogens have emerged as a result of a lack of rapid diagnostic tests with high sensitivity and specificity.

The majority of clinical microbiology laboratories still use the culture method for the detection of most bacteria from clinical samples; however, this process takes days to weeks to complete, relies on phenotypic biochemical characterization and requires skilled laboratory staff [3,4]. Apart from that, antibody detection methods have been used to detect circulating antibodies that are specific to respective bacteria; however, during acute infection, these results may be negative because the patients have not yet generated antibody response, and cross-reactions with unrelated IgM can occur [5]. Indeed, over the last two decades, there has been a surge in the development of diagnostic tests based on amplification and detection of specific bacterial nucleotide sequences. The majority of nucleic acid amplification methods use polymerase chain reaction (PCR) and can detect a pathogen of interest with high sensitivity and specificity; however, the requirement for expensive instruments (thermocycler) and reagents prevents such diagnostic tests from being used in areas with limited resources, such as on the battlefield or in developing countries [6]. As a result, the test cannot meet the ASSURED criteria (Affordable, Sensitive, Specific, User-friendly, Robust and rapid, Equipment-free, Deliverable) for developing point-of-care (POC) diagnostic tests [7].

Recently, enzymes from clustered, regularly interspaced, short palindromic repeats (CRISPR)- CRISPR associated protein (Cas) systems have been adapted for the specific, rapid, sensitive, and portable sensing of nucleic acids. The CRISPR–Cas system is composed of RNA-guided endonucleases, and it is an adaptive immune system that protects its hosts from bacteriophage predation and parasitism by other mobile genetic elements (MGEs) [8]. CRISPR-Cas system has been hailed as a versatile and reliable method for genome editing since its discovery. The CRISPR-Cas system has a diverse set of Cas proteins and genomic loci architecture, which has piqued researchers’ attention in a variety of biotech disciplines, including infectious disease detection [9]. Various CRISPR–Cas system-based methods have been developed for bacterial detection. However, there are limitations in terms of the number of comprehensive reports on successful bacterial detection using CRISPR–Cas technology. Therefore, this review aims to highlight the advances made in using the CRISPR-Cas systems to detect bacterial diseases.

## 2. Methods

The present scoping review utilized the updated Preferred Reporting Items for Systematic review and Meta-Analyses extension for Scoping Reviews (PRISMA-ScR) guidelines [10,11]. The PRISMA-ScR aims to provide guidance on the reporting of scoping reviews. This review has been registered in the International Prospective Register of Systematic Reviews (PROSPERO), with registration number CRD42021289140.

### 2.1. Search Strategy

The literature search was conducted in October 2021 through three databases (PubMed, Scopus, and Cochrane Library) using lists of keywords referring to the Medical Subject Headings (MeSH) thesaurus. These keywords were combined using the Boolean operators as follows: [“CRISPR”] AND [“bacteria”] AND [“detection”]. An additional search was conducted by manually screening the references of the retrieved literature.

### 2.2. Selection of Studies

Articles were excluded if (i) the studies did not involve the development and evaluation of CRISPR-based detection; (ii) the studies were published in languages other than English or Malay; (iii) the studies detected pathogens other than bacteria. The retrieved literature was downloaded into the Endnote reference manager and duplicates were identified and removed. The references were distributed to two authors (K.S. and M.A.N.), who independently reviewed all the articles for title and abstract screening. A satisfactory agreement for the screening process was assessed between the authors. Discrepancies between the authors were solved through a discussion with a third author (M.F.K.). Two authors (K.S. and M.A.N.) performed full-text screening and summarized the findings.

### 2.3. Questions for the Quality Assessment of Retrieved Studies

The studies included from PubMed, Scopus and Cochrane Library databases were analysed according to their quality standards by seven questions defined by the Critical Appraisal Skills Programme (CASP) qualitative checklist. For each retrieved article, the questions were answered by two authors who filled in an Excel table with the answers “no”, “yes” or “unclear”. Discrepancies between these authors were solved through a discussion with third authors. The questions are listed as follows: 1. Was there a clear statement of the aims of the research? 2. Was the research design appropriate to address the aims of the research? 3. Was the execution of the index test described in sufficient detail to permit replication of the test? 4. Did the study provide a clear definition of what was considered to be a positive result? 5. If necessary, have ethical issues been taken into consideration? 6. Was the data analysis sufficiently rigorous? 7. Is there a clear statement of findings?

### 2.4. Data Extraction

The following data were extracted for descriptive analysis and comparison in terms of percentage: type of bacteria, type of CRISPR-Cas enzymes, the occurrence of trans-cutting, extraction utilization, amplification methods, detection techniques, type of targets, assay time, and limit of detection (LoD).

## 3. Results

### 3.1. Search Results

**Figure 1:**
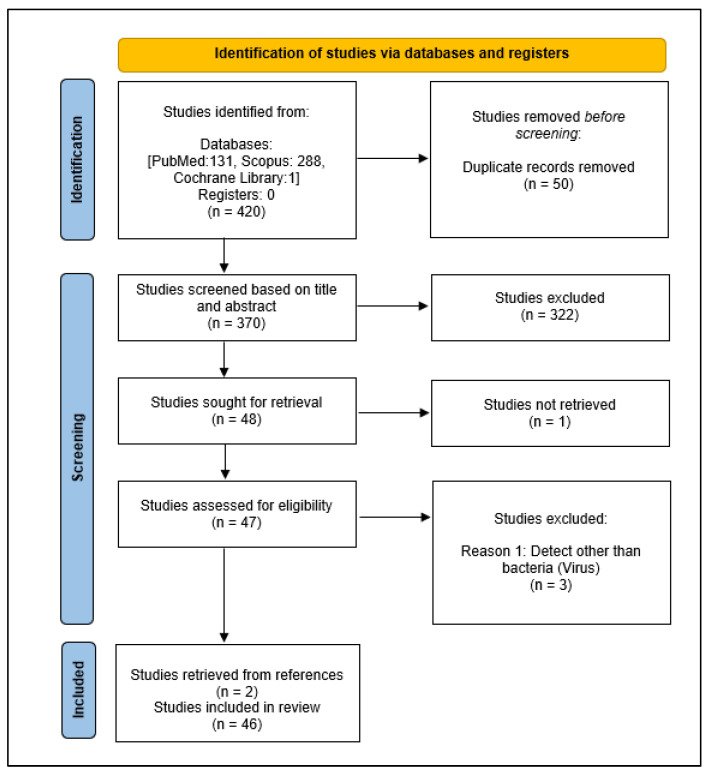
PRISMA-ScR flow diagram showing the process of selecting studies.

A total of 420 studies were identified from the three databases and 50 duplicates were removed. After screening the titles and abstracts, 322 studies that are not relevant were excluded. One article was not retrieved. Based on the study criteria, three studies were excluded during full-text screening. A total of two new studies were identified through manual searches of the lists of references. The remaining 46 studies were included in the final review (Figure 1). The characteristics of the 46 studies were summarized in Table 1. 

**Figure 2:**
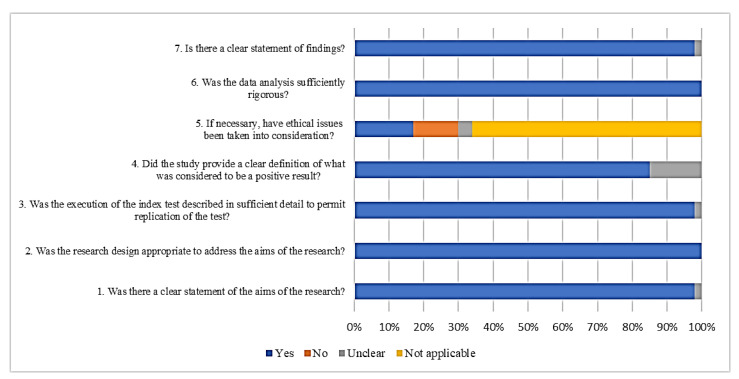
Quality assessment of the retrieved studies.

A summary of the CASP Qualitative Checklist assessment is presented in Figure 2. The overall results of the quality assessment showed a low risk of bias in all 46 studies. Regarding the answers for each quality question, around 2% of the studies did not clearly state the aims of the research, but all the studies showed an appropriate experimental design (100%). Most of the studies (98%) explained the index test in detail. When we analysed the definition of a positive result for those studies testing diagnostic approaches, 15% of the studies failed to report a cut off value. Thirteen percent of the studies did not consider ethical issues when biological samples were used. All studies (100%) reported sufficiently rigorous analyses of their data. Most of the studies (98%) displayed their findings clearly.

### 3.2. Types of Bacteria

**Figure 3:**
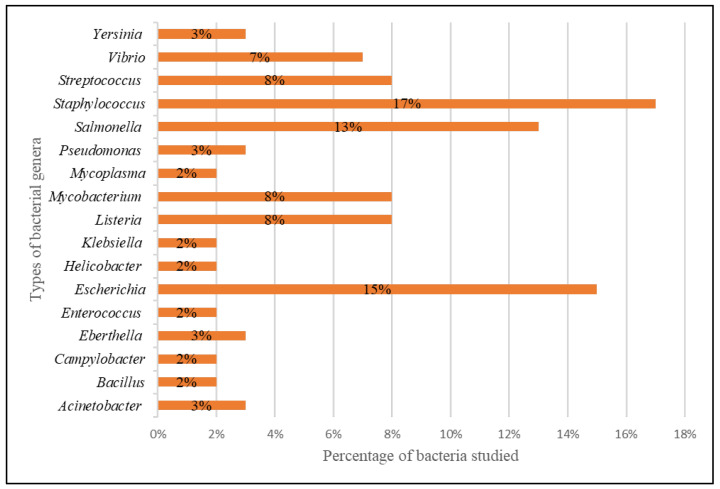
Bacteria from various genera were detected via CRISPR-Cas systems.

All 46 studies reported the development of diagnostics based on the CRISPR-Cas systems for the detection of harmful bacteria. The diagnostic platform has detected bacteria from 17 different genera, as shown in Figure 3. Most of the bacteria discovered in the retrieved studies came from the genera *Staphylococcus* (17%), *Escherichia* (15%), *Salmonella* (13%), *Listeria* (8%), *Mycobacterium* (8%) and *Streptococcus* (8%).

### 3.3. Detection Techniques

Table 2 showed a subgroup analysis of CRISPR-Cas-based-bacterial detection. Subgroup analysis based on CRISPR-Cas enzymes showed that Cas12a (64%) is the most commonly employed Cas enzyme in bacterial detection, followed by Cas13a (13%), and Cas9 (11%). Among 46 studies, there are several orthologs of Cas12a (LbCas12a, EnGen^®^ LbaCas12a, LbaCas12a and FnCas12a), Cas13 (LbuCas13a, LwaCas13a and LwCas13a) and Cas9 (dCas9). Cas12b, Cas14a, and dCas9 were utilised in an equal number of studies (4% each). The number of studies that employed trans-cleavage activity of CRISPR-Cas enzymes was higher (83%) and the remaining 17% of the studies used signalling molecules such as Raman reporter (methylene blue) and SYBR Green 1 because these studies predominantly used dCas9 which lacks trans-cleavage activity.

The extraction step is crucial to isolate bacterial genetic materials before amplification and CRISPR-Cas systems-based detection. Eighty-three percent of research employed an extraction process, while only 17% did not. Rather than isolating their genetic components, most of this research used aptamers, which are specific for selected bacteria that were amplified subsequent steps before being detected using the CRISPR-Cas systems. Subgroup analysis regarding the amplification method showed that 66% of the studies used isothermal reactions such as RCA, RPA, LAMP, SDA, EXPAR and RAA followed by PCR (23%) and amplification-free (11%). In terms of detection methods, the majority of research employed fluorescence (67%) based detection rather than lateral flow biosensor (13%), electrochemical biosensor (11%), and others (9%) such as surface-enhanced Raman scattering (SERS), gel electrophoresis, and colorimetric assay.

### 3.4. Types of Biomarkers

**Figure 4:**
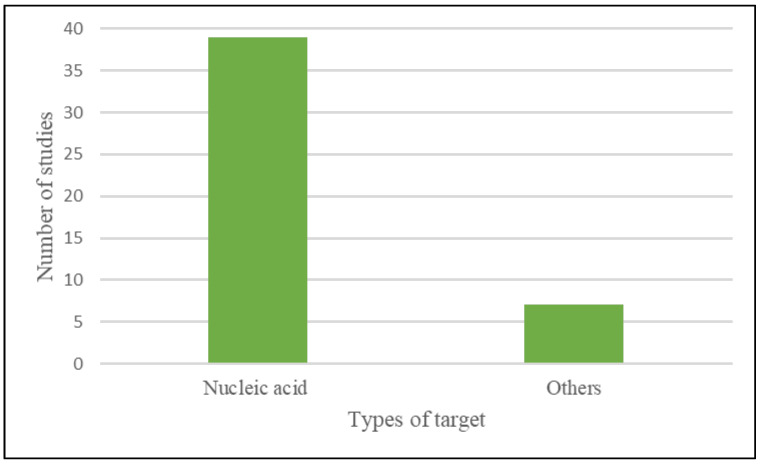
Types of targets used in CRISPR-Cas system-based bacterial detection.

The majority of research (39) employed nucleic acid as a target, while seven used non-nucleic acid such as aptamers and antibodies that are specific for the bacteria being detected as shown in Figure 4.

### 3.5. Assessment of Study Outcomes

Of the 46 studies, the turnaround times were 30 min to 4 h. The CRISPR-mediated DNA-FISH for the detection of Methicillin-resistant Staphylococcus aureus (MRSA) showed the fastest turnaround times of 30 min [44]. The rapid turnaround time was attributed to the amplification free method utilizing the dCas9 enzyme. Three studies did not report the turnaround times of the assays [30,45,56]. With regards to the analytical sensitivity of the CRISPR-Cas assays, the limit of detection (LoD) was evaluated in three types of concentration which include copies per mL, CFU per mL and molarity. For studies reporting the LoD in copies per mL, detection of Mycobacterium tuberculosis H37Rv utilizing EnGen LbaCas12a reported the lowest LoD of 1 copy/uL [40]. Meanwhile, most of the studies reported the LoD in CFU/mL, of which seven studies showed LoD of 1 CFU/mL. Five studies reported the LoD in molarity [12,17,18,28,38,39]. Of these, CAS-EXPAR for the detection of Listeria monocytogenes showed the lowest LoD up to the attomolar level [38]. Three studies did not report the LoD of the assays [42,48,55]. To determine the LoD of the target, most studies used spiked samples (78%) compared to clinical samples (22%).

## 4. Discussion

The CRISPR-Cas system has been used in various applications such as gene editing, identification of genotypes and SNPs, detection of antibiotics resistance and virulence genes, and diagnosis of infectious diseases [58,59,60]. Diagnostic techniques based on the CRISPR-Cas systems have recently attracted the attention of researchers due to their excellent accuracy. Three key aspects of the CRISPR-Cas systems contribute to high sensitivity and specificity in the diagnosis of disease, including the detection of bacteria. First, CRISPR– Cas systems identify specific amplicon sequences, distinguish them from amplification byproducts, and cut the sequences (cis-cleavage), a single-turnover method that improves specificity. Second, multiple turnover trans-cleavage activity of Cas12 and Cas13 causes nucleic acid signalling reporters to be cleaved several times, resulting in amplified readout signals for detection and hence improved sensitivity. Third, CRISPR–Cas systems make it easier to generate a variety of readout signals, which broadens their usefulness [61,62].

Bacteria from the genera *Staphylococcus* (17%), *Escherichia* (15%), *Salmonella* (13%), *Listeria* (8%), *Mycobacterium* (8%) and *Streptococcus* (8%) were mostly recognised by the CRISPR-Cas systems. Apart from that, the CRISPR-Cas systems were also utilised to detect bacteria from the genera (i) *Vibrio* (7%), (ii) *Yersinia* (3%), (iii) *Pseudomonas* (3%), (iv) *Eberthella* (3%), (v) *Acinetobacter* (3%), (vi) *Bacillus* (2%), (vii) *Campylobacter* (2%), (viii) *Enterococcus* (2%), (ix) *Helicobacter* (2%), (x) *Klebsiella* (2%), and (xi) *Mycoplasma* (2%). The majority of these bacteria are microbes responsible for the most common foodborne infections [63]. These pathogens are also antibiotic resistance bacteria and are on the World Health Organization (WHO)’s priority list for new antibiotic research and development [64,65]. A report by the United States Center for Disease Control and Prevention (CDC) provides an overview of the annual morbidity and mortality of antibiotic-resistant infections in the United States, estimating their number at approximately 2.8 million and the number of deaths associated with these infections at 35,000 [66].

The Cas enzyme is an endonuclease that may be programmed to detect DNA and RNA. CRISPR-Cas systems are divided into two classes (Class 1 and Class 2). Class 1 employs a multi-subunit crRNA-Cas protein, whereas Class 2 employs a single multidomain crRNA-Cas protein [67]. Class 2 is only found in bacteria, and accounts for less than 5% of all known systems. Each class has at least three types as well as several subtypes [68]. In Class 1, there are three types: I, III, and IV. Class 2 enzymes include the II (Cas9), V (Cas12), and VI (Cas13) enzyme classes, as well as subtypes such as V-A (Cas12a or Cpf1), V-B (Cas12b or C2c1), V-C (Cas12c or C2c3), V-F (Cas12f), VI-A (Cas13a or C2c2), VI-B (Cas13b or C2c4), VI-C (Cas13c or C2c7) and VI-D (Cas13d), which have evolved in separate evolutionary paths [69,70].

Of the 46 studies, 30 (64%) employed Cas12a enzymes to identify bacteria, while six (13%) studies used Cas13a, and five (11%) studies used Cas9. Cas12a has two major benefits over other Cas enzymes, namely, it does not require an additional reverse-transcription of amplicons step to detect bacterial DNA as compared to Cas13a [71], and it has trans-cleavage activity to non-specifically cut reporter probes to improve sensitivity, whereas Cas9 can only serve as a nickase to cut dsDNA (cis-cleavage) with no indication of trans-cleavage [62]. According to the Table 1, there are several Cas12a and Cas13a orthologs compared to Cas9, such as LbCas12a, EnGen^®^ LbaCas12a, LbaCas12a (*Lachnspiraceace* bacterium ND2006), FnCas12a (*Francisella novicida* U112), LbuCas13a (*Leptotrichia buccalis*), and LwaCas13a and LwCas13 (*Leptotrichia wadei*). Cas enzyme orthologs are important because they can recognise other protospacer adjacent motifs (PAM) sequences in addition to typical PAM sequences, allowing for a wider targeting range while maintaining target specificity [72].

Cas12b, Cac12f, and dCas9 were utilised in a similar number of studies (4% each). Recently, researchers have shown interest in a new ortholog Cas12b (AapCas12b) because this enzyme (from *Alicyclobacillus acidiphilus*) can tolerate high temperature (60 °C) of isothermal reaction (e.g., LAMP) compared to Cas12a, which operates at a lower temperature (e.g., 25–40 °C), and so is incompatible with high-temperature conditions and leads to one-pot assays [73,74]. As well as AapCas12b, BrCas12b from (thermophile bacterium *Brevibacillus* sp. SYSU G02855) is also capable of binding and cleaving target DNA at high temperatures, making it a good candidate for diagnostic development [75]. Apart from Cas12b, Cas14a (Cas12f) is becoming more widely utilised in the diagnostic field because it demands full complementarity in the seed region of sgRNA, a trait that is important for obtaining single nucleotide specificity [76]. Cas12f, a type V effector protein, was previously known as Cas14. Cas14 is similar to Cas12 in that it can also target dsDNA and is dependent on T-rich PAM, hence it has been classed into the Cas12 family (Karvelis et al., 2020). Cas9 from *Streptococcus pyogenes* (SpCas9) is one of the simplest systems, drawing a lot of attention for its gene-editing capabilities [77]. However, in recent years, it has become a good bio-recognition element after being modified in a deactivated form (dCas9) resulting in an “antibody-like” mechanism. Researchers created a dCas9 by introducing two-point mutations, H840A and D10A, into the HNH and RuvC nuclease domains (dCas9). DNA cleavage activity is absent in dCas9, but DNA binding activity is unaffected [78].

Furthermore, Class 2 Cas enzyme selection is based on the enzymes’ properties. Cas9 (type II) has two nuclease domains, HNH and RuvC, which each cleaves one strand of double-stranded DNA (dsDNA) [79]. Cas12 (type V) has only one RuvC domain that cleaves dsDNA and single-stranded DNA (ssDNA) in the presence of cation ions such as magnesium and calcium ions (Mg^2+^ and Ca^2+^) [80]. Cas13 (type VI) has two predicted higher eukaryotic and prokaryotic nucleotide (HEPN) domains to cut single-stranded RNA (ssRNA) [81]. Cas9 requires both tracrRNA and crRNA, whereas Cas12 (except for Cas12b and Cas12c) and Cas13 utilise crRNA only. This is because Cas12 and Cas13 can cleave crRNA arrays to produce their crRNAs (self-processing) [81,82].

Cas9 recognise a specific PAM sequence (5′ NGG 3′) (N represents any nucleotide) in a non-target DNA strand, distant 10–12 nucleotides apart from the PAM sequence [79]. Cas12 identifies a 5′-T-rich PAM at the distal end. The PAM sequence required for Cas12 to bind dsDNA induces the catalytic activation of a crRNA-complementary dsDNA, but not of a crRNA-complementary ssDNA [83]. Cas13a identifies a 3′ end non-G protospacer flanking site (PFS) while 5′ end non-C PFS for Cas13b [84,85]. Moreover, Cas9 generate a blunt dsDNA break while Cas12 and Cas13 generate sticky and near U and A break respectively [80,86]. In addition, Cas9 does not exhibit trans-cleavage activity, but Cas12 and Cas13 do, allowing for powerful signal amplification [87,88]. These discoveries result in the development of the DETECTR (DNA endonuclease targeted CRISPR trans reporter) and SHERLOCK (Specific High Sensitivity Enzymatic Reporter UnLOCKing) diagnostic platforms for Cas12 and Cas13 respectively [89,90]. Trans-cleavage activity by Cas12a and Cas13 were used in 83% of studies, while the remaining 17% of studies mostly used Cas9, which lacks trans-cleavage activity and hence used other signalling molecules.

Extracting bacterial genetic materials from samples necessitates determining the appropriate nucleic acid sequence (DNA or RNA) [91]. It is an important preanalytical stage in the development of any successful molecular diagnostic procedure, ensuring a reliable result. The extraction method was first used in 83% of studies before continuing to amplify and before CRISPR-Cas system-based detection. Extraction entails lysing the cells, purifying the nucleic acid to remove extraneous cell components, inhibiting compounds, degrading enzymes, and recovering the necessary nucleic acid [92,93]. The commercially available kits are also used for DNA extraction.

To improve sensitivity, the CRISPR-Cas system needs to be combined with a target nucleic acid amplification step performed by either PCR or isothermal technologies. This allows for the enrichment of rare and low-abundance nucleic acid targets and for the depletion of unwanted abundant nucleic acids. PCR is used in terms of targeted amplification of desired sequences, but it has several disadvantages, including the need for large equipment and trained personnel to operate it [94]. Isothermal amplification technologies address these constraints. CRISPR–Cas9 systems are effective for creating isothermal exponential amplification strategies because they can unwind dsDNA to ssDNA at a moderate temperature (37 °C) [61]. Isothermal methods were used in 66% of the investigations, while PCR methods were used in 23% of the studies. Among isothermal methods, RPA has been widely used in CRISPR-Cas system based bacterial detection followed by LAMP and SDA. RPA is notable for its ease of use, high sensitivity, selectivity, compatibility with multiplexing, exceptionally rapid amplification (20–60 min), and ability to operate at a low (37–42 °C) and constant temperature without the requirement for an initial denaturation phase or numerous primers [59,95]. The amplification technique, on the other hand, is not only time consuming but also poses a risk of aerosol contamination. Several research groups have worked hard to produce amplification-free CRISPR-Cas systems and investigate their use in pathogen identification. Only five (11%) of the 46 research studies found used an amplification-free CRISPR-Cas system-based bacterial detection.

Fluorescence detection (67%), lateral flow biosensor (13%), and electrochemical biosensor (11%) are three key detection approaches employed in CRISPR-Cas systems-based bacteria detection. Fluorescence-based sensing has several advantages, including background-free sensing, which dramatically improves the signal-to-noise ratio when compared to other optical approaches, but which necessitates the use of instruments (fluorescence reader, either portable or not, and a real-time PCR machine) [96]. The main benefit of LFAs is that their results are simple to interpret and can be read with the naked eye without the use of expensive instruments. LFAs, on the other hand, are inefficient and inaccurate, making these traditional paper-based platforms unsuitable for the higher quantitative analyses required in clinical applications [97]. Biosensors have several advantages over traditional analytical techniques, including excellent selectivity and sensitivity, the potential for miniaturization and portability, low cost, real-time detection, small sample volumes, and quick reaction [98].

## 5. Conclusions

This review detailed and analysed several key points, including the origin of CRISPR-Cas systems, the types and properties of CRISPR-Cas enzymes, extraction methods, amplification techniques, detection methods, assay time and LoD. CRISPR-Cas systems were first used to modify genes and are now being used to diagnose infectious diseases. Cas12, Cas13, and Cas9 are three types of CRISPR-Cas enzymes employed in bacterial detection. Nucleic acid extraction prior to isothermal amplification, and fluorescence detection steps after CRISPR-Cas were used in most identified studies in this review. The assay takes about 4 h and obtains various LoD using spiked samples. This review reveals that only 20% of the studies reported on the clinical accuracy of the CRISPR-Cas system in bacterial detection. As a result, we recommend that the development and evaluation of the CRISPR-Cas system should be conducted using clinical samples. It is suggested that more studies will focus on the development of nucleic acid amplification-free CRISPR-Cas systems for bacterial detection toward the fulfilment of the ASSURED criteria.

## Figures and Tables

**Table 1:** Summary of the included studies.

No	CRISPR-Cas Enzymes	Name of Methods	Types of Bacteria	Trans Cutting	Reporter Probes	Amplification Methods	Extraction	Samples (Types & n)	Detection Methods	Requirements of Instruments	Targets	Assay Time (min)	LOD/ Detection Range	References
1	LbaCas12a	NR	*Escherichia coli* & *Staphylococcus aureus*	Yes	ssDNA	PCR	Yes	Reference laboratory strains & panel of clinical isolates	EB	Thermocycler & impedance analyser	*mdh* & *nuc*	90	3 nM	[12]
2	Cas9 Cis (dsDNA)	NR	*Escherichia coli* O157:H7	No	ssDNA with a metal-organic framework (MOF) [UiO66]	SDA & RCA	Yes	Spiked spring water, skim milk & orange juice	F	Thermocycler & fluorescence spectrophotometer	*hlyA*	120	40 CFU/mL	[13]
3	Cas12a	CRISPR-MTB	*Mycobacterium tuberculosis* H37Ra strain	Yes	ssDNA	RPA	Yes	Pulmonary samples: sputum, & BALF and extrapulmonary samples: cerebrospinal fluid, pleural fluid, ascites, pus, pericardial effusion, urine & synovial fluid (n = 179)	F	qPCR machine	IS6110	90	50 CFU/mL	[14]
4	LbuCas13a	APC-Cas	*Salmonella* enteritidis	Yes	ssRNA	SDA & reverse transcription	No	Spiked milk and drinking water	F	Real-time PCR machine	Aptamer SE-3 against live *Salmonella Enteritidis*	140	1 CFU	[15]
5	AacCas12b	TB-QUICK	*Mycobacterium tuberculosis* H37Ra strain	Yes	ssDNA	LAMP	Yes	Sputum, BALF & EDTA anticoagulant plasma (n = 147)	F	Real-Time PCR machine	IS6110	120	1.3 copy/μL	[16]
6	LbCas12a	NR	*Mycobacterium tuberculosis*	Yes	ssDNA	RPA	Yes	Sputum (n = 193)	F	Real-Time PCR machine	IS1081	240	4.48 fmol/L	[17]
7	dCas9	NR	*Staphylococcus aureus*, *Acinetobacter baumannii* & *Klebsiella pneumoniae*	No	Raman reporter: MB	No	Yes	Mice lung, spleen & liver tissues	SERS	Raman spectrometer	*spa*, *pgi* & *uge*	60	14.1 fM, 9.7 fM & 8.1 fM	[18]
8	Cas12a	CRISPR-HP	*Helicobacter pylori*	Yes	ssDNA	RPA	Yes	Stool (n = 41)	LFB	No	Genomic DNA	60	5 copies/µL	[19]
9	EnGen^®^ LbaCas12a	NR	Methicillin-resistant *Staphylococcus aureus* (MRSA)	Yes	ssDNA	SDA	No	Reference laboratory strains	F	Fluorescence spectrophotometer	Aptamer against PBP2a	~180	10^6^ to 10^2^ CFU/ mL (detection range)	[20]
10	Cas13a	CRISPR-GBS	*Streptococcus agalactiae*	Yes	ssRNA	RPA	Yes	Vaginal–rectal swabs (n = 412)	F	qPCR machine	*atoB*	90	50 CFU/mL	[21]
11	EnGen^®^ LbaCas12a	BCA–RPA–Cas12a	*Salmonella* typhimurium	Yes	ssDNA	RPA	No	Spiked milk	F	Blue light	Antibodies specific against *Salmonella Typhimurium*	60	1 CFU/mL	[22]
12	LbCas12a	OCTOPUS	*Escherichia coli* O157:H7 & *Streptococcus aureus*	Yes	ssDNA	RPA	Yes	Spiked milk	F	Portable fluorescence reader	*rfbE* & *nuc*	50	1 CFU/mL	[23]
13	Cas12a	NR	*Salmonella* typhimurium	Yes	ssDNA	LAMP	Yes	Inactivated bacterial culture	F	Portable UV lamp & milk warmer	Genomic DNA	60	800 CFU/mL	[24]
14	LbCas12a	RPA-Cas12a-FS	*Escherichia coli, Listeria monocytogenes, Staphylococcus aureus* & *Vibrio parahaemolyticus*	Yes	ssDNA	RPA	Yes	Pork, duck meat, and beef	F	Handheld fluorometer	Genomic DNA	45	10 copies (*Escherichia coli, Listeria monocytogenes, Staphylococcus aureus*) & 100 copies (*Vibrio parahaemolyticus*)	[25]
15	Cas9 Cis (dsDNA)	Cas9nAR	*Salmonella* typhimurium	No	SYBR Green I	Cas9nAR	Yes	Bacteria isolates	F	Fluorescence reader	*invA*	60	1 copy/10µL	[26]
16	LbaCas12a	CIA	*Pseudomonas aeruginosa*	Yes	ssDNA	LAMP	Yes	Spiked human serum, milk & clinical sputum	LFB	No	Acetyltransferase	50	1 CFU/mL	[27]
17	LbCas12a	NR	*Yersinia pestis*	Yes	ssDNA	RPA	Yes	Reference laboratory strains & clinical isolates	F & LFB	Real-time PCR machine	Chromosomal DNA (4 tag sites)	~50 (LFB)-150 (F)	10^3^ fg/µL (YP-1, YP-2 & YP-3)-10^6^ fg/µL (YP-4) [F]	[28]
18	Cas12f(a)	Cas-TSPE	*Escherichia coli, Eberthella typhi, Pseudomonas aeruginosa, Staphylococcus aureus, Streptococcus pyogenes* & *Enterococcus faecalis*	Yes	ssDNA	PCR	Yes	Spiked blood & urine	F	Fluorescence reader	Variable regions (V3) of 16S rRNA	~210	1 CFU/mL (*Streptococcus pyogenes*)	[29]
19	Cas12a	NR	*Escherichia coli* O157:H7	Yes	ssDNA	RCA	No	Spiked skimmed milk powder	EB	CHI660E electrochemical workstation & CE	Aptamer	NR	10 CFU/mL	[30]
20	LbaCas12a	NR	*Escherichia coli* O157:H7	Yes	ssDNA	Primer exchange reaction & SDA	No	Spiked milk	EB	CHI660E electrochemical workstation & CE	Aptamer	~180	19 CFU/mL	[31]
21	LbCas12a	NR	*Salmonella* spp.	No. Catalyze TMB-H_2_O_2_ reaction (Blue to yellow)	G-quadruplex hemin (DNAzyme)	RPA	Yes	Spiked beer & juice	Colorimetric & quantitative analysis	Smartphone readout with Color Picker APP	*invA*	180	1 CFU/mL	[32]
22	AacCas12b	NR	*Campylobacter jejuni*	Yes	ssDNA	PCR	Yes	Spiked chicken (n = 55)	F	Thermal Cycler & blue light	*flhA*	40	10 CFU/g	[33]
23	EnGen^®^ LbaCas12a	NR	Gram-negative bacteria: *Escherichia coli* (LPS)	Yes	ssDNA	No	No (purchased LPS)	Spiked purified water, milk, grapefruit juice & green tea	F (inhibitory effect)	Fluorescence spectrometer	Aptamer	~140	23 CFU/mL	[34]
24	Cas9 Cis (dsDNA) nickase	NR	*Salmonella* typhimurium & *Escherichia coli*	No	ssDNA (fluorescence tagged Primers)	Cas9nAR	Yes	Spiked milk	LFB	Portable test strip reader	*invA* & *UidA*	180	100 CFU/mL	[35]
25	LbCas12a	NR	*Staphylococcus aureus*	Yes	ssDNA	PCR	Yes	Spiked milk	Elementary OR AND INHIBIT logic gates	Microplate reader	*femA*	120	10^3^ CFU/mL	[36]
26	Cas9	CASLFA	*Listeria monocytogenes*	No	AuNP-DNA Probe	PCR	Yes	Reference laboratory strains	LFB	Thermocycler	*hlyA*	60	150 copies	[37]
27	Cas9 Cis (ssDNA)	CAS-EXPAR	*Listeria monocytogenes*	No	SYBR Green I	EXPAR	Yes	Bacterial cells	F	Real-time PCR	*hly*	60	0.82 amol	[38]
28	Cas12a	E-Si-CRISPR	Methicillin-resistant *Staphylococcus aureus* (MRSA)	Yes	ssDNA	No	Yes	Spiked human serum	EB	PGSTAT204 AutoLab, SPGE & impedance analyser	*mecA*	90	3.5 fM	[39]
29	EnGen^®^ LbaCas12a	NR	*Mycobacterium tuberculosis* H37Rv	Yes	ssDNA	RPA	Yes	BALB, hydrothorax, and homogenate of needle biopsy (n = 69)	Gel electrophoresis	No	IS6110	40	1 copy/uL	[40]
30	LwCas13a	PCF	*Salmonella* spp.	Yes	ssRNA	PCR & reverse transcription	Yes	Reference laboratory strains & bacterial isolates	F	Thermocycler & fluorescence reader	*invA*	120	10 CFU/mL	[41]
31	FnCas12a	NR	*Mycobacterium abscessus* species and subspecies	Yes	ssDNA	PCR	Yes	Clinical isolates	F	Thermocycler & Fluorescence reader	*rpoB & erm* (41)	~240	NR	[42]
32	LbaCas12a	NR	Multidrug-resistant *Acinetobacter baumannii* (MDRAB)	Yes	ssDNA	PCR	Yes	Reference laboratory strains	F	Thermocycler & fluorescence spectrophotometer	*glt A* & β-lactamase genes	120	50 CFU/mL	[43]
33	dCas9	CRISPR-mediated DNA-FISH	Methicillin-resistant *Staphylococcus aureus* (MRSA)	No	SYBR Green I	No	Yes	Bacterial cells	F	Fluorescence spectroscopy	*mecA*	30	10 CFU/mL	[44]
34	Cas13a	NR	*Bacillus cereus*	Yes	Light-up RNA aptamer (Broccoli)	No	Yes	Spiked milk & rice	F	Fluorescence microplate reader	16s rRNA	NR	9.83 CFU	[45]
35	LwCas13a	CCB-Detection	*Staphylococcus aureus*	Yes	ssRNA	PCR & reverse transcription	Yes	Spiked milk, juice, beer & water	F	Microplate reader	*nuc*	240	1 CFU/mL	[46]
36	LbCas12a	NR	*Salmonella* spp.	Yes	ssDNA	PCR	Yes	Spiked milk	F	Portable colorimeter & portable NIR irradiator	*invA*	90	1 CFU/mL	[47]
37	Cas12a	NR	Methicillin resistant *Staphylococcus aureus* (MRSA)	Yes	ssDNA	RCA	No	Spiked serum	F	Fluorescence spectroscopy	Aptamers against protein A & PBP2a	~75	NR	[48]
38	LwaCas13a	SHERLOCK	*Yersinia pestis*	Yes	ssRNA	RPA	Yes	Reference laboratory strains & bacterial isolates	F	Microplate reader	*lcrV*	150	420 copies/mL	[49]
39	Cas12a	NR	*Vibrio parahaemolyticus*	Yes	ssDNA	PCR	Yes	Spiked shrimp	F	Homemade UV light & mini thermal cycler	*tlh*	~100	102 copies/μL	[50]
40	LbCas12a	Cas12a-UPTLFA	*Yersinia pestis*	Yes	ssDNA	RPA	Yes	Spiked blood	LFB	UPT biosensor	*pla*	80	1 CFU/µL	[51]
41	EnGen^®^ LbaCas12a	RAA-based E-CRISPR	*Listeria monocytogenes*	Yes	ssDNA with methylene blue	RAA	Yes	*Flammulina velutipes*	EB	CHI 660E electrochemical workstation & gold electrodes	*LMOSLCC2755_0090*	120	26 CFU/mL	[52]
42	EnGen^®^ LbaCas12a	Cas12aFDet	*Listeria monocytogenes* serotype 4c	Yes	ssDNA	RAA	Yes	Spiked fresh grass carp	F	Fluorescence reader	*LMOSLCC2755_0090*	60	135 CFU/mL	[53]
43	Cas12a	NR	*Vibrio parahaemolyticus*	Yes	ssDNA	LAMP	Yes	Spiked shrimp	F	Portable cartridge	*tlh*	50	30 copies/reaction	[54]
44	EnGen^®^ LbaCas12a	Cas12aVDet	Mycoplasma	Yes	ssDNA	RPA	No	Cell culture supernatant	F	Blue light	16s rRNA	30	NR	[55]
45	Cas12f(a1)	CMP	*Streptococcus pyogenes* & *Eberthella typhi*	Yes	ssDNA	Reverse-transcription & APCR	Yes	Spiked milk	F	Fluorescence plate reader	16S rRNA gene V3 hypervariable region	NR	10^3^ & 10^4^ CFU/mL	[56]
46	Cas12a	RAA-CRISPR/Cas12a	*Vibrio vulnificus*	Yes	ssDNA	RAA	Yes	Spiked human blood & stool	F	UV torch	*vvhA*	40	2 copies/reaction	[57]

**Table 2:** Summary of subgroup analysis of CRISPR-Cas system-based-bacterial detection.

Subgroup	Number of Studies in Percentage
All studies	46 (100%)
**Extraction of nucleic acids**	
Yes	83%
No	17%
**Amplification of nucleic acids**	
Isothermal reaction	66%
Polymerase chain reaction	23%
No	11%
**CRISPR-Cas enzymes**	
Cas12a	64%
Cas12b	4%
Cas13a	13%
Cas12f	4%
Cas9	11%
dCas9	4%
**Trans-cleavage activity**	
Yes	83%
No	17%
**Detection methods**	
Fluorescence	67%
Lateral flow biosensor	13%
Electrochemical biosensor	11%
Others	9%

## Data Availability

Not applicable.
